# Trends in Mortality from Septicaemia and Pneumonia with Economic Development: An Age-Period-Cohort Analysis

**DOI:** 10.1371/journal.pone.0038988

**Published:** 2012-06-14

**Authors:** Irene O. L. Wong, Benjamin J. Cowling, Gabriel M. Leung, C. Mary Schooling

**Affiliations:** 1 Lifestyle and Life Course Epidemiology Group, School of Public Health, Li Ka Shing Faculty of Medicine, The University of Hong Kong, Hong Kong SAR, China; 2 CUNY School of Public Health at Hunter College, New York, New York, United States of America; Swiss Tropical & Public Health Institute, Switzerland

## Abstract

**Background:**

Hong Kong population has experienced drastic changes in its economic development in the 1940s. Taking advantage of Hong Kong’s unique demographic and socioeconomic history, characterized by massive, punctuated migration waves from Southern China, and recent, rapid transition from a pre-industrialized society to the first ethnic Chinese community reaching “first world” status over the last 60 years (i.e., in two or three generations), we examined the longitudinal trends in infection related mortality including septicemia compared to trends in non-bacterial pneumonia to generate hypotheses for further testing in other recently transitioned economies and to provide generalized aetiological insights on how economic transition affects infection-related mortality.

**Methods:**

We used deaths from septicemia and pneumonia not specified as bacterial, and population figures in Hong Kong from 1976–2005. We fitted age-period-cohort models to decompose septicemia and non-bacterial pneumonia mortality rates into age, period and cohort effects.

**Results:**

Septicaemia-related deaths increased exponentially with age, with a downturn by period. The birth cohort curves had downward inflections in both sexes in the 1940s, with a steeper deceleration for women. Non-bacterial pneumonia-related deaths also increased exponentially with age, but the birth cohort patterns showed no downturns for those born in the 1940s.

**Conclusion:**

The observed changes appeared to suggest that better early life conditions may enable better development of adaptive immunity, thus enhancing immunity against bacterial infections, with greater benefits for women than men. Given the interaction between the immune system and the gonadotropic axis, these observations are compatible with the hypothesis that upregulation of the gonadotropic axis underlies some of the changes in disease patterns with economic development.

## Introduction

With economic development and epidemiological transition there are large increases in life expectancy as deaths from infectious diseases decline and are only partially offset by increases in deaths from the chronic diseases of affluence. This decline in infectious diseases is normally conceptualized in terms of better living conditions and public health measures, such as vaccination, sanitation and access to health care, affecting contemporaneous vulnerability and exposure to infections with corresponding effects on mortality. However, an important hypothesis about the history of evolution and ageing is that humans, like other animals, work with a limited resource base and trade-off certain life-history parameters against each other [1]. According to this hypothesis, less limited early life conditions may be embodied in a constitution designed to maximize both long-term survival, via resistance to infections, and reproductive success [2]. Moreover, given the differing investments in reproduction by sex, there may be sex-specific developmental strategies enabled by less constrained environments [3], [4], where women may invest more in immuno-competence, and men more in sexual dimorphism and sexual success [3], via androgens with associated long-term detrimental consequences for adaptive immune function [5], [6].

Few studies have examined whether an environment enabling optimal growth and development confers long-term resistance to infectious diseases [7], [8], [9], [10], [11], or whether there are the same benefits for men and women. Moreover, this question may not be answerable in commonly studied Western populations where economic transition took place over generations and the range of early living conditions may be insufficient to have marked effects on the development of the immune system. However, that does not preclude potential relevance to the majority of the global population living in developing countries where there is a much wider range of early living conditions.

We took advantage of a unique population with a well-characterized history of macro environmental events including economic transition over one life time with a drastic change in living conditions at a defined historical time. The Hong Kong population was formed by mass migration of young people looking for work from pre-industrial Southern China to comparatively developed Hong Kong, following world war II, in the mid 20^th^ century [12], where, all experienced rapid economic development, albeit with temporary uncertainty in the 1990s before the return of Hong Kong to China and the Asian financial crisis in 1997. Nevertheless, despite relatively high levels of inequality, Hong Kong has transitioned to a post-industrial economy with a social infrastructure equivalent to other developed countries, including publicly funded hospital care open to all, which was strengthened by the establishment of a sole public provider (the Hospital Authority) in 1990. Currently the Hong Kong population has good access to care and one of the longest life expectancies in the world, but different lifetime experiences by cohort. The pre-1940s birth cohorts were born into and grew up in very limited living conditions, whilst the post 1940s birth cohorts were born into a substantially more developed environment. In our study, we investigated the longitudinal trends in infection related mortality including septicemia compared to trends in non-bacterial pneumonia deaths. We decomposed septicemia mortality rates into the effects of age, period and cohort and examined whether the effects varied by sex. We investigated septicemia-related mortality, because resistance to bacterial infections is likely to be most closely linked to adaptive immunity [13] and thereby may change disease patterns in the population. To indicate specificity, we also similarly considered death from pneumonia not specified as bacterial, as it is an infectious disease that may be caused by a wider range of organisms [14], be the sequelae to other illnesses or may simply represent the consequences of senescence [15]. Thus, in this paper, we examined longitudinal trends in mortality due to bacterial-related disease, i.e., septicemia, and due to pneumonia not-specified as bacteria-related in the Hong Kong population in order to describe the potential effects of economic transition on mortality risk due to infections and to generate hypotheses for further testing in other recently transitioned economies as well as to provide generalized aetiological insights.

## Methods

### Sources of Data

We obtained data on mortality due to septicaemia (ICD 9=38, ICD10= A40, A41, A021, A227, A267, A327) and pneumonia not specified as bacterial (ICD9=480, 485–486; ICD10= J12, J12.0, J12.1, J12.2, J12.8, J12.9, J16, J16.8, J17, J17.1, J17.8, J18 and J18.9) (we called pneumonia hereafter) and mid-year population figures for the years 1976 to 2005 from the Hong Kong Death Registry and the Census and Statistics Department respectively. The Hong Kong Death Registry is a population-based registry covering the registered deaths in the respective years for the entire local resident population. We included all deaths associated with septicaemia and pneumonia related deaths during the period of observation in the analysis. We grouped the death and population data into fourteen 5-year age groups from 20–24 years to 85 or above, and six 5-year calendar time periods from 1976–1980 to 2001–2005 respectively. This classification resulted in nineteen birth cohorts centred at 5-year intervals beginning in the calendar year 1896.

### Statistical Analysis

We modelled septicemia- and pneumonia-related deaths by using Poisson age-period-cohort (APC) models [16] which decomposes death rates over time by chronological age, calendar period and birth cohort. The modelling framework has previously been used to study secular trends for chronic diseases [17], [18] and also for infectious diseases [19].

In the model, we chose the second and the penultimate periods (1981–1985 and 1996–2000) and the central birth cohort (1936–41) as the reference categories [17], [18]. We used Bayesian inference to estimate the model parameters [20]. Because of the identifiability problem of APC models [16], only second-order changes (i.e., changes in slopes or inflection points) are interpretable. We assessed whether these second-order changes coincided with the timing of defined macro environmental events during the economic transition in Hong Kong over last century [17], [18].

We specified second-order Gaussian autoregressive priors in the forward direction for the age, period and cohort effects [20]. These priors specified that the initial expected value of each effect was based on an extrapolation from its two immediate predecessors. We estimated the model parameters using Markov Chain Monte Carlo (MCMC) simulations with 5 concurrent chains started at different initial values since comparison of multiple chains can allow us to discern convergence. We used the criteria R-hat to monitor convergence [20]. Based on the values of R-hat, we discarded the first 10,000 samples as a burn-in period, and then took a further 40,000 samples from the posterior distributions. The parameter estimates and derived rates were summarised in terms of posterior means and 95% credible intervals.

The model goodness-of-fit was measured by the posterior mean deviance 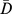 [21]_._ To compare models, the deviance information criterion (DIC) was calculated, which adjusts the posterior mean deviance for the number of parameters in the model [21]. A smaller DIC implies a better fit. To examine whether the trends differed by sex, we looked for any potential difference in age, cohort or period effects by sex assessed from the model fit using the DIC. To make any comparisons by sex explicit and interpretable [22], we included men and women in the same model thus obtaining joint estimates relative to a common reference point of men for each component, i.e., men in 1981–1985 and 1996–2000 for the period effect, and men born 1936–41 for the birth cohort effect. Divergence between the sexes indicates different associations by sex. All analyses were implemented using R version 2.10.1 (R Development Core Team, Vienna, Austria) and WinBUGS version 1.4 (MRC Biostatistics Unit, Cambridge, UK).

## Results

### Age-standardized Mortality Rates due to Septicemia and Pneumonia

**Figure 1:**
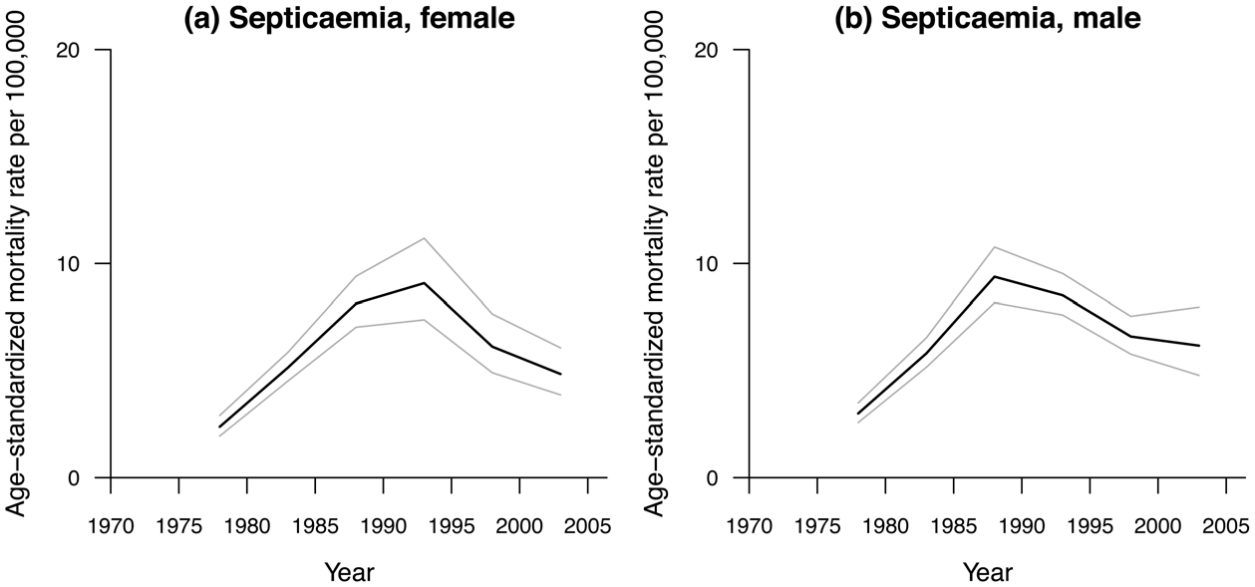
Age-standardized mortality rates due to septicemia per 100,000 in Hong Kong, 1975–2005 for (a) female, (b) male populations.

**Figure 2:**
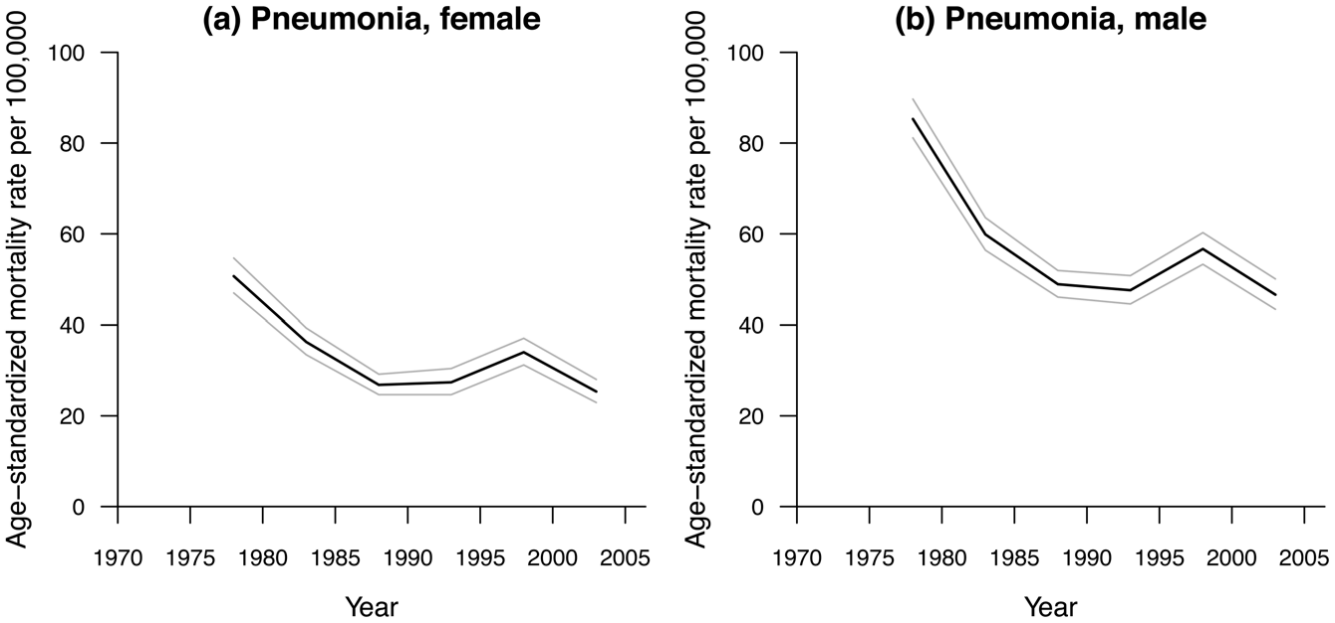
Age-standardized mortality rates due to pneumonia per 100,000 in Hong Kong, 1975–2005 for (a) female, (b) male populations.

Figures 1 and 2 show the observed sex-specific age-standardized mortality rates for septicemia and pneumonia in Hong Kong from 1976–2005, respectively. Septicemia mortality rates showed an inverted U-shape, which peaked around 1985 to 1995 where female curve peaks later than male’s one (∼1995 vs ∼1985). Pneumonia death rates showed an overall downward trend but had upward short-term increases around 1998. Mortality rates were higher for men than women.

### Age, Period and Birth Cohort Effects by Sex

**Figure 3:**
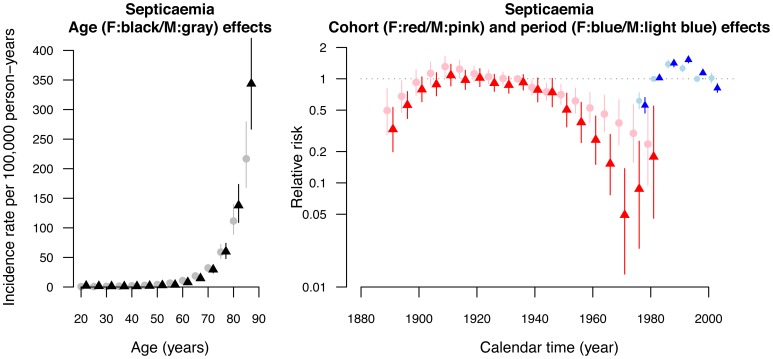
Parameter estimates of age, period and cohort effects from the age-period-cohort model (DIC=1157.3) with sex-age, sex-cohort and sex-period interactions (solid circle points for male and triangle points for females). Left hand panel: Estimated age-specific annual male mortality rates due to septicaemia (ICD 9=38, ICD10= A40, A41, A021, A227, A267, A327) in 5-year age groups in Hong Kong with 95% credible intervals. Right hand panel: Estimated relative risks for 10-year birth cohorts (nineteen birth cohorts beginning in the calendar year 1896 for each sex) and 5-year calendar periods with 95% credible intervals (six periods beginning from 1976 for each sex).

**Figure 4:**
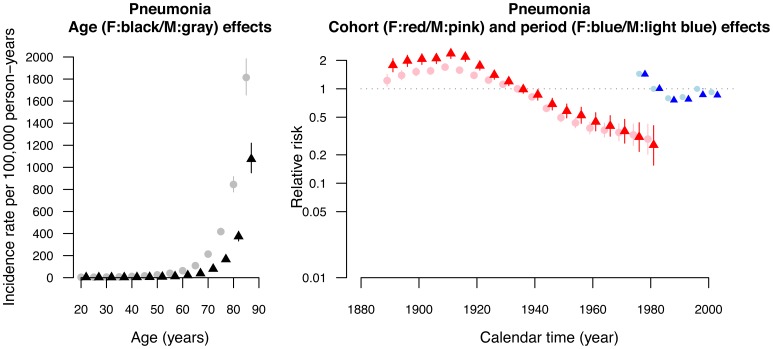
Parameter estimates of age, period and cohort effects from the age-period-cohort model (DIC=1486.8) with sex-age, sex-cohort and sex-period interactions (solid circle points for male and triangle points for females). Left hand panel: Estimated age-specific annual female mortality rates due to pneumonia (ICD9=485–486; ICD10= J12 to J18) in 5-year age groups in Hong Kong with 95% credible intervals. Right hand panel: Estimated relative risks for 10-year birth cohorts (nineteen birth cohorts beginning in the calendar year 1986 for each sex) and 5-year calendar periods with 95% credible intervals (six periods beginning from 1976 for each sex).

We fitted age, period and cohort effects sequentially and compared different models in terms of DICs. Age, period and cohort all contributed to both septicemia and pneumonia mortality rates, and varied with sex, as assessed from the DIC (Table S1). The two full APC models with sex interactions provided the best fit with the smallest values of DICs compared to the other partial models (Table S1). There was also a downturn for the cohort effect for both septicemia and pneumonia in about 1910 (Figure 3, Figure 4). It coincided with the collapse of the Qing dynasty (ending centuries of successive dynastic rule in 1911) and a subsequent fall in living standards, due to the ensuing instability from civil strife. This is most likely a systematic effect due to the population history, whereby any pre-1911 birth cohorts among the mid 20^th^ century migrants had already exceeded life expectancy in China, and were with age increasingly strongly selected healthy migrants [17], [18].

Septicemia-related deaths increased exponentially with age in both sexes (Figure 3). There was a down turn in the period effects in both sexes, earlier in men than women. The birth cohort curves had notable downward inflections in both sexes in about 1940, with a steeper deceleration in women after 1945s (Figure 3). There was also an upward inflection in the birth cohort effect for women in about 1970 with relatively wide credibility intervals. In contrast, although pneumonia related deaths also increased exponentially in both sexes, the period and birth cohort patterns for pneumonia-related deaths were different from those for septicemia (Figure 4). There was an upturn for the period effect in the mid 1980s. There was no downward inflections in the birth cohort curve in the 1940s or 1950s; instead there was an upward inflection in about 1950.

## Discussion

There were birth cohort-driven decreases in mortality due to septicemia, which were stronger for women than men. In contrast, we did not identify the mid 20^th^ century birth cohort effect on the changes in non-bacterial related pneumonia deaths, and patterns in pneumonia did not differ by sex. We, therefore, hypothesize that drastic improvement in the living conditions of the general population over a short period of time during the mid 20^th^ century appeared to have exerted differing birth cohort effects on deaths due to septicemia and non-bacterial related pneumonia, albeit such differences may provide etiological insight rather than having a large effect on overall mortality rates. More specifically, there was negative noticeable change/inflection of birth cohort curves for the septicemia while less apparent change for pneumonia-related one.

Previous studies have examined overall trends in septicemia. These have generally found increasing trends in bacteremia related diseases [23], [24], which have been ascribed to improvements over decades in diagnosis and greater use of more complex interventions [23], [25]. We similarly observed an increasing trend in septicemia mortality rates in the earlier part of the period (Figure 1), which eventually began to decrease. However, to our knowledge there are no previous studies which have decomposed septicemia mortality into the effects of age, period and cohort or examined early influences on subsequent vulnerability to septicemia in older adults. There are some indications that poor early life conditions may be associated with death from infections [11], but these studies have been too small to examine differences by sex. Generally, poor early life conditions are usually positively associated with inflammatory markers in adults [26], [27], [28], with similar differences by sex in resource poor settings, whereby better early living conditions are more strongly negatively associated with inflammation among women than men [27], [29]. Thus our observations are somewhat consistent with previous studies.

Despite these intriguing results some potential pitfalls should be noted. First, our study is descriptive and we can only speculate about the etiologies of the changes observed. Differences in decline between cohorts may be explained by the other etiological and epidemiological factors other than hormonal-related one. However, APC analysis can be particularly valuable in developing or recently developed populations, where other sources of information or long-running cohort studies, may be lacking. Also, we examined whether any aspects of the model could be the artefactual results of data changes, such as coding changes or other specific events that have taken place during the period. Second, we do not have the information to incorporate potential factors and mediators such as education or lifestyle into these models. However, we found very different patterns for septicemia and pneumonia, which suggests that the observed patterns cannot both be the result of uncontrolled co-variable adjustment. Third, the number of cases of septicemia was relatively low. Nevertheless, the downturn for the mid 20th century birth cohorts was very clear with a visible separation between the sexes in the second half of the 20^th^ century. Moreover, we have refrained from any interpretation of the upturn, with relatively wide credibility intervals, for the most recent cohorts of women. However, we could not eradicate the possibility that the upward inflection might not be apparent for the birth cohort with wide credibility intervals for septicemia after 1970. Other possible hypothesis could be the influence of adaptive immunity would convey more resistance to septicemia deaths among older adult women. Further investigation and re-analysis when more deaths have accumulated in the more recent cohorts would therefore be needed. Fourth, women may be less likely to develop sepsis but more likely to die as a consequence of sepsis, which although it is not inconsistent with our observations and hypothesis, and has implications for the proposed pathway. Nevertheless, the changes in the pattern of septicemia-related deaths could be attributed by changes in prevalence of co-morbid diseases including diabetes or infecting organism, or an increase in nosocomial sepsis (as opposed to community-acquired). Fifth, the age standardized rates for septicemia increased and decreased during the period, as reflected in the period effects. There have been general changes in medical technology and practices, specifically improvements in the ability to detect bacteremia [23], [25], and more intensive interventions [30], which likely resulted in increasing trends in septicaemia [23], [24]. Whereas more specifically in Hong Kong, the establishment of the Hospital Authority in 1990, thereby making a milestone in the system-wide upgrade of inpatient service delivery, may have improved access to care and standardized cause of death coding. Moreover, one might argue that time trends would be influenced by the periodic financial events, transition of the local administrative governments and the establishment of the Hospital Authority with better inpatient delivery infrastructure. But these incidents would be expected not to influence secular trends differently for deaths due to septicemia and pneumonia. Thus, we are also cautious in our interpretation of the period effects. However, for these contemporaneous population wide changes to have affected the sex-specific birth cohort effects, they would need to represent changes in medical practice which were applied differentially by age and sex, which is unlikely. Moreover, the shift from the classification of ICD9 to ICD10 may not affect the quality and consistency of our grouping for the mortality data due to septicemia and pneumonia. Despite a higher level of specificity in ICD10 regarding the conditions that resulted in the deaths, the non-bacterial pneumonia and septicemia death data would be coded based on two coding systems. But, we cannot rule out the possibility that the septicemia deaths in particular could be sometimes underreported as a major cause of death. Sixth, among patients admitted with pneumonia, it would be possible that the underlying organism cannot be identified, and of these, patients could be due to viral in origin. Seventh, the introduction of pneumococcal, meningococcal or influenza vaccination programmes in older people or infants and children could have affected deaths from septicemia or pneumonia either directly or via herd immunity. However, pneumococcal and meningococcal vaccination programmes were introduced into Hong Kong after the study period, whilst influenza vaccination was introduced at the end of the study period, targeted at the elderly living in residential care homes in 1998 and more generally in 2003–4. As such, it is unlikely that introduction of influenza vaccine materially impacted cohort effects for septicemia or pneumonia.

There are several possible interpretations of our findings. Our observations for septicemia could reflect reduced population mixing for succeeding generations since about 1940, which particularly affected women, driven by forces such as changing family sizes, family responsibilities and working patterns. If that were the case we might have expected similar patterns for pneumonia which were not evident. Moreover, there were no consistent birth cohort effects for deaths due to septicemia and non-bacterial pneumonia. This might suggest that changes in the disease risks could not be fully accounted only by the well-acknowledged factors such as improved access to health care, changes in diagnostic practices and medical advancement, because these would generally be expected to drive population wide period effects rather than generate sex and disease specific birth cohort effects. Another possibility is different psychosocial responses by gender either to population wide events, such as the Asian financial crisis, or at sensitive developmental phases. Gender specific responses to population wide events would be expected to generate sex-specific period effects, which were not evident. However, gender specific psychosocial responses at sensitive developmental phases, such during childhood or puberty, could potentially have gender specific effects on long-term development of the immune system.

Alternatively, we hypothesize that early life conditions may have long-term biological sex-specific effects on the functioning of specifically the adaptive immune system. The thymus plays a key role in the development of adaptive immunity, because it is where the lifetime repertoire of T cells is created [31]. The thymus is most active before puberty [31] and is sensitive to malnutrition, micro-nutrient deficiencies and infections during growth and development [32], [33], [34]. Levels of sex-steroids at puberty are environmentally driven, for example by nutrition or stress, in animals [35], [36] and humans [37], [38], with potentially life-long consequences. Experimental and other evidence indicates that adaptive immune function is promoted by estrogen and suppressed by testosterone [6], [39]. Overall, better living conditions in early life would be expected to promote adaptive immunity, with greater effects among women, consistent with the observed cohort effects for septicemia. These findings are compatible with our hypothesis that upregulation of the gonadotropic axis with better early living conditions may obscure some of the protective effects of social advantage among men [4]. However, we cannot prove the underlying biological pathways in this study.

Clearly these observations need to be replicated in other suitable settings. From a public health perspective these observations potentially suggest that promoting optimal growth and development could enhance adaptive immune function, although some of the gains among men may be partially offset by higher levels of androgens enabled by the same environments. Whether better adaptive immunity would also be expected to protect against aging [40], and thereby also relate to the greater gains in life expectancy among women than men with economic development remains to be determined.

## Supporting Information

Table S1 **Deviance information criterion (DIC) values (correct to four significant figures) for different combinations for age, period and cohort models for bacteria-related deaths due to septicemia and pneumonia in Hong Kong.**
(DOC)

## References

[1] Johnston SL, Grune T, Bell LM, Murray SJ, Souter DM (2006). Having it all: historical energy intakes do not generate the anticipated trade-offs in fecundity.. Proceedings of the Royal Society Biological Sciences Series B.

[2] van Bodegom D, May L, Meij HJ, Westendorp RG (2007). Regulation of human life histories: the role of the inflammatory host response.. Annals of the New York Academy of Sciences.

[3] Schooling CM, Jiang CQ, Zhang W, Lam TH, Cheng KK, et al (2011). Size does matter: adolescent build and male reproductive success in the Guangzhou Biobank Cohort Study.. Annals of Epidemiology.

[4] Schooling CM, Hui LL, Ho LM, Lam TH, Leung GM (2011). Cohort Profile: ‘Children of 1997’: a Hong Kong Chinese birth cohort.. International Journal of Epidemiology.

[5] Grossman CJ (1985). Interactions between the gonadal steroids and the immune system.. Science.

[6] Shames RS (2002). Gender differences in the development and function of the immune system.. Journal of Adolescent Health.

[7] McDade TW (2005). Life history, maintenance, and the early origins of immune function.. American Journal of Human Biology.

[8] Martins VJ, Toledo Florencio TM, Grillo LP, Franco MC, Martins PA (2011). Long-lasting effects of undernutrition.. International Journal of Environmental Research and Public Health.

[9] Hall AJ, Yee LJ, Thomas SL (2002). Life course epidemiology and infectious diseases.. International Journal of Epidemiology.

[10] Cheng AC, West TE, Limmathurotsakul D, Peacock SJ (2008). Strategies to reduce mortality from bacterial sepsis in adults in developing countries.. PLOS Medicine.

[11] Moore SE, Collinson AC, Tamba NP, Aspinall R, Prentice AM (2006). Early immunological development and mortality from infectious disease in later life.. Proceedings of the Nutrition Society.

[12] Tsang S (2004). A modern history of Hong Kong: Hong Kong: Hong Kong University Press..

[13] Opal SM, Girard TD, Ely EW (2005). The immunopathogenesis of sepsis in elderly patients.. Clinical Infectious Diseases.

[14] Falsey AR, Walsh EE (2006). Viral pneumonia in older adults.. Clinical Infectious Diseases.

[15] Janssens JP, Krause KH (2004). Pneumonia in the very old.. Lancet Infectious Diseases.

[16] Holford TR (1991). Understanding the effects of age, period, and cohort on incidence and mortality rates.. Annual Reviews of Public Health.

[17] Wong IOL, Cowling BJ, Schooling CM, Leung GM (2007). Age-period-cohort projections of breast cancer incidence in a rapidly transitioning Chinese population.. International Journal of Cancer.

[18] Wong IOL, Cowling BJ, Law SCK, Mang OWK, Schooling CM, et al (2010). Understanding socio-historical imprint on cancer risk by age-period-cohort decomposition in Hong Kong.. Journal of Epidemiology and Community Health.

[19] Wu P, Cowling BJ, Schooling CM, Wong IOL, Johnston JM, et al (2008). Age-period-cohort analysis of tuberculosis notifications in Hong Kong from 1961 to 2005.. Thorax.

[20] Bray I (2002). Application of Markov chain Monte Carlo methods to projecting cancer incidence and mortality.. Journal of the Royal Statistical Society Series C (Applied Statistics).

[21] Speigelhalter DJ, Best NG, Carlin BP, van der Linde A (2002). Bayesian mesaures of model complexity and fit.. Journal of the Royal Statistical Society: Series B (Statistical Methodology).

[22] Rosenberg PS, Anderson WF (2010). Proportional hazards models and age-period-cohort analysis of cancer rates.. Statistics in Medicine.

[23] Sogaard M, Engebjerg MC, Lundbye-Christensen S, Schonheyder HC (2011). Changes in blood culture methodology have an impact on time trends of bacteraemia: a 26-year regional study.. Epidemiology and Infection.

[24] Pittet D, Wenzel RP (1995). Nosocomial bloodstream infections. Secular trends in rates, mortality, and contribution to total hospital deaths.. Archives of Internal Medicine.

[25] Madsen KM, Schonheyder HC, Kristensen B, Sorensen HT (1999). Secular trends in incidence and mortality of bacteraemia in a Danish county 1981–1994.. APMIS.

[26] Loucks EB, Pilote L, Lynch JW, Richard H, Almedia N (2010). Life Course Socioeconomic Position Is Associated With Inflammatory Markers: The Framingham Offspring Study.. Social Science and Medicine.

[27] Nazmi A, Oliveira IO, Horta BL, Gigante DP, Victora CG (2010). Lifecourse socioeconomic trajectories and C-reactive protein levels in young adults: findings from a Brazilian birth cohort.. Social Science and Medicine.

[28] Schooling CM, Jiang C, Lam TH, Zhang W, Cheng KK, et al (2011). Parental Death during Childhood and Adult Cardiovascular Risk in a Developing Country: The Guangzhou Biobank Cohort Study.. PLoS One.

[29] Schooling CM, Jiang CQ, Lam TH, Zhang WS, Cheng KK, et al (2011). Childhood meat eating and inflammatory markers: The Guangzhou Biobank Cohort Study.. BMC Public Health.

[30] Steinberg JP, Clark CC, Hackman BO (1996). Nosocomial and community-acquired Staphylococcus aureus bacteremias from 1980 to 1993: impact of intravascular devices and methicillin resistance.. Clinical Infectious Diseases.

[31] Lynch HE, Goldberg GL, Chidgey A, Van den Brink MR, Boyd R (2009). Thymic involution and immune reconstitution.. Trends in Immunology.

[32] Hollander GA, Krenger W, Blazar BR (2010). Emerging strategies to boost thymic function.. Current Opinion in Pharmacology.

[33] Savino W, Dardenne M, Velloso LA, Dayse Silva-Barbosa S (2007). The thymus is a common target in malnutrition and infection.. British Journal of Nutrition 98 S11-S16.

[34] McDade TW, Beck MA, Kuzawa CW, Adair LS (2001). Prenatal undernutrition and postnatal growth are associated with adolescent thymic function.. Journal of Nutrition.

[35] Adam CL, Findlay PA (1997). Effect of nutrition on testicular growth and plasma concentrations of gonadotrophins, testosterone and insulin-like growth factor I (IGF-I) in pubertal male Soay sheep.. Journal of Reproduction and Fertility.

[36] Slob AK, Vreeburg JT, Van der Werff ten Bosch JJ (1979). Body growth, puberty and undernutrition in the male guinea-pig.. British Journal of Nutrition.

[37] Dorgan JF, Hunsberger SA, McMahon RP, Kwiterovich PO, Lauer RM (2003). Diet and sex hormones in girls: findings from a randomized controlled clinical trial.. Journal of National Cancer Institute.

[38] Campbell BC, Gillett-Netting R, Meloy M (2004). Timing of reproductive maturation in rural versus urban Tonga boys, Zambia.. Annals of Human Biology.

[39] Choudhry MA, Bland KI, Chaudry IH (2006). Gender and susceptibility to sepsis following trauma.. Endocrine Metabolic Immune Disorders.

[40] Sauce D, Larsen M, Fastenackels S, Duperrier A, Keller M (2009). Evidence of premature immune aging in patients thymectomized during early childhood.. The Journal of Clinical Investigation.

