# Supplementary material for: Trends in Mortality from Septicaemia and Pneumonia with Economic Development: An Age-Period-Cohort Analysis
Source: PLoS One. 2012 Jun 14;7(6):e38988. doi: 10.1371/journal.pone.0038988 (PMC3375224; doi:10.1371/journal.pone.0038988)
Supplement: Table S1 — Deviance information criterion (DIC) values (correct to four significant figures) for different combinations for age, period and cohort models for bacteria-related deaths due to septicemia and pneumonia in Hong Kong. (DOC) [file pone.0038988.s001.doc]

Table S1. Deviance information criterion (DIC) values (correct to four significant figures) for different combinations for age, period and cohort models for bacteria-related deaths due to septicemia and pneumonia in Hong Kong

| Model | Components | Cause of death | |
| --- | --- | --- | --- |
|  |  | Septicemia | Pneumonia |
| 1 | Age | 2508 | 13220 |
| 2 | Period | 44200 | 312900 |
| 3 | Cohort | 11880 | 67330 |
| 4 | Age, period | 1637 | 10190 |
| 5 | Age, cohort | 1968 | 10200 |
| 6 | Age, period, cohort | 1394 | 8764 |
| 7 | Age*sex, period, cohort | 1195 | 1604 |
| 8 | Age, period*sex, cohort | 1325 | 4740 |
| 9 | Age, period, cohort*sex | 1258 | 2533 |
| 10 | Age, period*sex, cohort*sex | 1223 | 2347 |
| 11 | Age*sex, period*sex, cohort | 1222 | 1539 |
| 12 | Age*sex, period, cohort*sex | 1193 | 1497 |
| 13 | Age*sex, period*sex, cohort*sex | 1157 | 1490 |
